# 2-Chloro-5-nitro­pyridine

**DOI:** 10.1107/S1600536810008974

**Published:** 2010-03-17

**Authors:** Seik Weng Ng

**Affiliations:** a Department of Chemistry, University of Malaya, 50603 Kuala Lumpur, Malaysia

## Abstract

The non-H atoms of the title compound, C_5_H_3_ClN_2_O_2_, almost lie in a common plane (r.m.s. deviation = 0.090 Å). In the crystal, adjacent mol­ecules feature a short Cl⋯O contact [3.068 (4) Å], forming a chain; these chains are consolidated into a layer structure by non-classical C—H⋯O inter­actions.

## Related literature

For the mechanism of the reaction between 2-chloro-5-nitro­pyridine and aryl­oxide ions, see: El-Bardan (1999 ▶); Haynes & Pett (2007 ▶); Zeller *et al.* (2007 ▶).
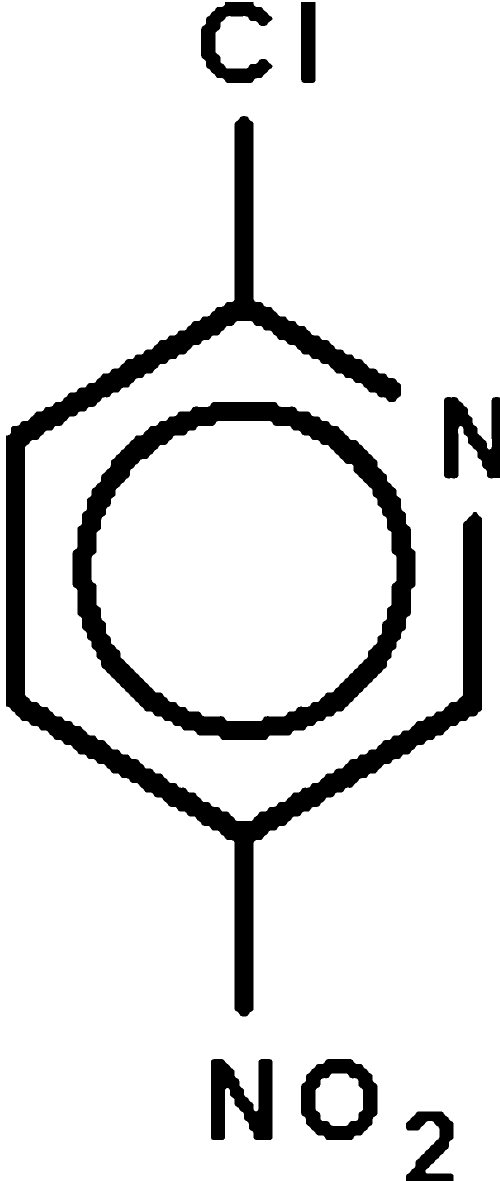


## Experimental

### 

#### Crystal data

C_5_H_3_ClN_2_O_2_
*M* *_r_* = 158.54
Triclinic, 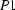

*a* = 3.7599 (8) Å
*b* = 5.8641 (13) Å
*c* = 7.0189 (15) Å
α = 84.687 (3)°
β = 89.668 (3)°
γ = 76.020 (3)°
*V* = 149.50 (6) Å^3^
*Z* = 1
Mo *K*α radiation
μ = 0.56 mm^−1^
*T* = 100 K
0.45 × 0.15 × 0.03 mm

#### Data collection

Bruker SMART APEX diffractometer
Absorption correction: multi-scan (*SADABS*; Sheldrick, 1996 ▶) *T*_min_ = 0.786, *T*_max_ = 0.983
1379 measured reflections
1114 independent reflections
1071 reflections with *I* > 2σ(*I*)
*R*_int_ = 0.022

#### Refinement

*R*[*F* ^2^ > 2σ(*F* ^2^)] = 0.056
*wR*(*F* ^2^) = 0.143
*S* = 1.17
1114 reflections
91 parameters
3 restraints
H-atom parameters constrained
Δρ_max_ = 0.63 e Å^−3^
Δρ_min_ = −0.59 e Å^−3^
Absolute structure: Flack (1983 ▶), 449 Friedel pairs
Flack parameter: −0.05 (14)

### 

Data collection: *APEX2* (Bruker, 2009 ▶); cell refinement: *SAINT* (Bruker, 2009 ▶); data reduction: *SAINT*; program(s) used to solve structure: *SHELXS97* (Sheldrick, 2008 ▶); program(s) used to refine structure: *SHELXL97* (Sheldrick, 2008 ▶); molecular graphics: *X-SEED* (Barbour, 2001 ▶); software used to prepare material for publication: *publCIF* (Westrip, 2010 ▶).

## Supplementary Material

Crystal structure: contains datablocks global, I. DOI: 10.1107/S1600536810008974/bt5214sup1.cif

Structure factors: contains datablocks I. DOI: 10.1107/S1600536810008974/bt5214Isup2.hkl

Additional supplementary materials:  crystallographic information; 3D view; checkCIF report

## Figures and Tables

**Table 1:** Hydrogen-bond geometry (Å, °)

*D*—H⋯*A*	*D*—H	H⋯*A*	*D*⋯*A*	*D*—H⋯*A*
C2—H2⋯O1^i^	0.95	2.50	3.361 (7)	151

Symmetry code: (i) 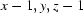.

**Fig. 1.**
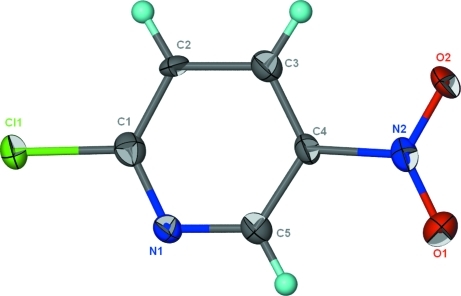
Anisotropic displacement ellipsoid plot (Barbour, 2001) of 2-chloro-5-nitropyridine at the 70% probability level; hydrogen atoms are drawn as spheres of arbitrary radius.

## supplementary crystallographic information

### Comment

We have synthesized some nitropyridyl aryl ethers by the reaction of the aryloxide ion with the chlorine-substituted nitropyridine. The mechanism of this reaction has been reported (El-Bardan, 1999). With 2-chloro-5-nitropyridine, additional hydroxide base should not be used as the compound undergoes ring opening (Haynes & Pett, 2007; Zeller *et al.*, 2007).

2-Chloro-5-nitropyridine (Scheme I, Fig. 1) is a flat molecule; the non-hydrogen atoms all lie in a common plane (r.m.s. deviation 0.090 Å). Adjacent molecules interact by a Cl···O contact [3.068 (4) Å] to form a chain. The chains are consolidated into a layer structure by a non-classical C–H···O interaction; this interaction involves the second oxygen atom of the nitro group.

### Experimental

2-Chloro-5-nitropyridine as supplied by Aldrich Chemical Company is crystalline.

### Refinement

H-atoms were placed in calculated positions (C—H 0.95 Å) and were included in the refinement in the riding model approximation, with *U*(H) set to 1.2*U*(C).

The checking program *PLATON* detects some pseudo symmetry. However, as the Flack parameter refined to nearly zero, the non-centric space group must be the correct one. Nevertheless, an attempt was made to treat the structure as a whole-molecule-disordered structure but this gave a model with bad bond dimensions.

### Crystal data

**Table:**

C_5_H_3_ClN_2_O_2_	*Z* = 1
*M**_r_* = 158.54	*F*(000) = 80
Triclinic, *P*1	*D*_x_ = 1.761 Mg m^−^^3^
Hall symbol: P 1	Mo *K*α radiation, λ = 0.71073 Å
*a* = 3.7599 (8) Å	Cell parameters from 637 reflections
*b* = 5.8641 (13) Å	θ = 2.9–28.2°
*c* = 7.0189 (15) Å	µ = 0.56 mm^−^^1^
α = 84.687 (3)°	*T* = 100 K
β = 89.668 (3)°	Plate, colorless
γ = 76.020 (3)°	0.45 × 0.15 × 0.03 mm
*V* = 149.50 (6) Å^3^	

### Data collection

**Table:**

Bruker SMART APEX diffractometer	1114 independent reflections
Radiation source: fine-focus sealed tube	1071 reflections with *I* > 2σ(*I*)
graphite	*R*_int_ = 0.022
ω scans	θ_max_ = 27.5°, θ_min_ = 2.9°
Absorption correction: multi-scan (*SADABS*; Sheldrick, 1996)	*h* = −4→4
*T*_min_ = 0.786, *T*_max_ = 0.983	*k* = −7→7
1379 measured reflections	*l* = −9→9

### Refinement

**Table:**

Refinement on *F*^2^	Secondary atom site location: difference Fourier map
Least-squares matrix: full	Hydrogen site location: inferred from neighbouring sites
*R*[*F*^2^ > 2σ(*F*^2^)] = 0.056	H-atom parameters constrained
*wR*(*F*^2^) = 0.143	*w* = 1/[σ^2^(*F*_o_^2^) + (0.070*P*)^2^ + 0.2047*P*] where *P* = (*F*_o_^2^ + 2*F*_c_^2^)/3
*S* = 1.17	(Δ/σ)_max_ = 0.001
1114 reflections	Δρ_max_ = 0.63 e Å^−^^3^
91 parameters	Δρ_min_ = −0.59 e Å^−^^3^
3 restraints	Absolute structure: Flack (1983), 449 Friedel pairs
Primary atom site location: structure-invariant direct methods	Flack parameter: −0.05 (14)

### Fractional atomic coordinates and isotropic or equivalent isotropic displacement parameters (Å^2^)

**Table:**

	*x*	*y*	*z*	*U*_iso_*/*U*_eq_	
Cl1	0.5000 (2)	0.50003 (18)	0.49998 (17)	0.0193 (3)	
O1	0.8088 (11)	0.9486 (7)	1.2731 (6)	0.0254 (9)	
O2	0.3611 (12)	1.2366 (7)	1.1607 (7)	0.0259 (10)	
N1	0.7126 (12)	0.5558 (8)	0.8407 (7)	0.0161 (10)	
N2	0.5729 (13)	1.0408 (10)	1.1526 (7)	0.0170 (11)	
C1	0.5156 (14)	0.6688 (11)	0.6894 (8)	0.0180 (11)	
C2	0.3197 (14)	0.9038 (9)	0.6723 (8)	0.0137 (11)	
H2	0.1837	0.9735	0.5592	0.016*	
C3	0.3320 (17)	1.0316 (12)	0.8277 (10)	0.0182 (13)	
H3	0.2017	1.1922	0.8260	0.022*	
C4	0.5415 (15)	0.9162 (10)	0.9860 (8)	0.0146 (11)	
C5	0.7254 (14)	0.6800 (10)	0.9887 (8)	0.0173 (11)	
H5	0.8644	0.6050	1.0995	0.021*	

### Atomic displacement parameters (Å^2^)

**Table:**

	*U*^11^	*U*^22^	*U*^33^	*U*^12^	*U*^13^	*U*^23^
Cl1	0.0220 (6)	0.0165 (6)	0.0198 (6)	−0.0033 (4)	−0.0008 (4)	−0.0072 (4)
O1	0.028 (2)	0.024 (2)	0.023 (2)	−0.0037 (17)	−0.0085 (18)	−0.0027 (18)
O2	0.034 (2)	0.015 (2)	0.024 (2)	0.0062 (16)	−0.0050 (17)	−0.0097 (17)
N1	0.015 (2)	0.014 (2)	0.019 (2)	−0.0011 (18)	−0.0014 (18)	−0.0041 (18)
N2	0.018 (2)	0.016 (3)	0.018 (3)	−0.005 (2)	0.002 (2)	−0.006 (2)
C1	0.016 (2)	0.019 (3)	0.020 (3)	−0.004 (2)	0.003 (2)	−0.005 (2)
C2	0.013 (2)	0.014 (2)	0.013 (3)	0.001 (2)	−0.0043 (18)	0.001 (2)
C3	0.017 (3)	0.016 (3)	0.021 (3)	−0.002 (2)	0.000 (2)	−0.004 (2)
C4	0.015 (2)	0.013 (2)	0.016 (3)	−0.003 (2)	0.0011 (19)	−0.005 (2)
C5	0.017 (2)	0.015 (3)	0.018 (3)	−0.003 (2)	0.001 (2)	−0.003 (2)

### Geometric parameters (Å, °)

**Table:**

Cl1—C1	1.739 (6)	C2—C3	1.387 (9)
O1—N2	1.219 (7)	C2—H2	0.9500
O2—N2	1.235 (7)	C3—C4	1.387 (9)
N1—C1	1.325 (7)	C3—H3	0.9500
N1—C5	1.330 (7)	C4—C5	1.389 (8)
N2—C4	1.455 (8)	C5—H5	0.9500
C1—C2	1.391 (7)		
			
C1—N1—C5	116.7 (5)	C2—C3—C4	117.6 (6)
O1—N2—O2	124.1 (6)	C2—C3—H3	121.2
O1—N2—C4	118.4 (6)	C4—C3—H3	121.2
O2—N2—C4	117.4 (5)	C3—C4—C5	120.8 (5)
N1—C1—C2	126.0 (5)	C3—C4—N2	120.5 (5)
N1—C1—Cl1	115.4 (4)	C5—C4—N2	118.7 (5)
C2—C1—Cl1	118.6 (4)	N1—C5—C4	122.0 (5)
C3—C2—C1	117.0 (5)	N1—C5—H5	119.0
C3—C2—H2	121.5	C4—C5—H5	119.0
C1—C2—H2	121.5		
			
C5—N1—C1—C2	−0.6 (7)	O1—N2—C4—C3	−166.8 (5)
C5—N1—C1—Cl1	−179.2 (4)	O2—N2—C4—C3	11.9 (9)
N1—C1—C2—C3	0.1 (8)	O1—N2—C4—C5	13.1 (8)
Cl1—C1—C2—C3	178.6 (4)	O2—N2—C4—C5	−168.1 (5)
C1—C2—C3—C4	0.8 (8)	C1—N1—C5—C4	0.1 (8)
C2—C3—C4—C5	−1.2 (8)	C3—C4—C5—N1	0.8 (8)
C2—C3—C4—N2	178.7 (4)	N2—C4—C5—N1	−179.2 (5)

### Hydrogen-bond geometry (Å, °)

**Table:**

*D*—H···*A*	*D*—H	H···*A*	*D*···*A*	*D*—H···*A*
C2—H2···O1^i^	0.95	2.50	3.361 (7)	151

Symmetry codes: (i) *x*−1, *y*, *z*−1.
